# Effect of manual physical therapy on sleep quality and jaw mobility in patients with bruxism: A biopsychosocial randomized controlled trial

**DOI:** 10.3389/fneur.2022.1041928

**Published:** 2022-12-08

**Authors:** Mohamed H. El-Gendy, Mostafa M. Ibrahim, Emad S. Helmy, Neama H. Neamat Allah, Batool Abdulelah Alkhamis, Ghada M. Koura, Hamada A. Hamada

**Affiliations:** ^1^Department of Basic Science, Faculty of Physical Therapy, Cairo University, Giza, Egypt; ^2^Department of Oral and Maxillofacial Surgery, Faculty of Oral and Dental Medicine, Cairo University, Giza, Egypt; ^3^Department of Biomechanics, Faculty of Physical Therapy, Cairo University, Giza, Egypt; ^4^Department of Medical Rehabilitation Sciences, College of Applied Medical Sciences, King Khalid University, Abha, Saudi Arabia; ^5^Department of Physical Therapy for Musculoskeletal Disorders and its Surgery, Faculty of Physical Therapy, Cairo University, Giza, Egypt

**Keywords:** bruxism, deep-stripping massage, pressure release massage, Pittsburgh Sleep Quality Index, manual physical therapy

## Abstract

This study aimed to investigate the effects of deep-stripping and trigger-point pressure release massage on the Pittsburgh Sleep Quality Index (PSQI), jaw mobility, and pressure pain threshold (PPT) of masticatory muscles in patients with sleep bruxism. A randomized controlled trial was conducted among 45 patients diagnosed with sleep bruxism. The patients were randomly assigned to three groups. Group I was the control group and included five men and 10 women; Group II was the deep-stripping massage group, which included two men and 13 women; and Group III was the pressure release group, which involved four men and 11 women. Patients were tested two times, before and after 6 weeks. Group I received transcutaneous electrical nerve stimulation and passive stretching; Group II received a deep-stripping massage; and Group III received a trigger-point pressure release massage. Findings revealed significant improvements in PSQI (*p* = 0.0001), jaw opening (*p* = 0.0001), jaw protrusion (*p* = 0.0001), jaw left lateral movement (*p* = 0.004), jaw retraction (*p* = 0.0001), right temporalis PPT (*p* = 0.0001), left temporalis PPT (*p* = 0.0001), right master PPT (*p* = 0.001), left master PPT (*p* = 0.001), right lateral pterygoid PPT (*p* = 0.001), left lateral pterygoid PPT (*p* = 0.001), right digastric muscle PPT (*p* = 0.001), and left digastric muscle PPT (*p* = 0.001) in the post-test condition in Group II compared with Group I and Group III. Deep-stripping massage improved PSQI, jaw mobility, or PPT of the masticatory muscles compared with trigger-point pressure release massage and traditional treatment techniques in patients with sleep bruxism.

## Introduction

Research into the neurological, sleep, and dental aspects of bruxism has lately gained traction. It manifests itself in two forms: awake and sleep bruxism. Awake bruxism manifests as a clenching habit of the jaw that develops in response to anxiety and stressful conditions (1). On the other hand, sleep bruxism manifests as a rhythmic masticatory activity related to sleep that is usually linked to arousals from sleep. Previous research showed that sleep bruxism is the most dangerous form, as it can cause damage to the teeth, periodontium, and oral mucosa; cause pathology of the masticatory muscles, cervical pain, headaches, hearing loss, and temporomandibular disorders (TMD) (2). Bruxism is also subclassified into primary and secondary bruxism. Primary bruxism is not associated with any other medical condition, while secondary bruxism is associated with neurological disorders or pharmacological side effects (3–5).

The diagnosis of bruxism is made clinically and based on the presence of typical signs and symptoms, including pain in the temporomandibular joint (TMJ), pain in the cervical and masticatory muscles, headache in the temporal zone when patients wake up in the morning, excessive tooth mobility, and poor sleep quality. Pain associated with bruxism has been thought to alter the recruitment of jaw muscles, which may result in a higher imbalance in such muscles and neuromuscular changes (6).

Previous studies supported the use of postural awareness, electrotherapy, therapeutic exercises, cognitive behavior therapy, muscle relaxation technique, massage therapy, and acupuncture to improve pain and jaw mobility in patients with bruxism. However, there is little support for the effectiveness of these treatments in the literature (7). Deep-stripping massage is often used along with other manual techniques to treat fibrositis (8). It is one of many medical massage techniques that have been thought to inhibit active trigger points and improve muscle length and circulation by targeting a specific band of muscle fibers (9).

To the best of the authors' knowledge, no research has investigated deep-stripping massage's effect on sleep quality, jaw mobility, or PPT in patients with bruxism. Therefore, the main purpose of this study was to examine the effect of deep-stripping massage on sleep quality, jaw mobility, or PPT of the masticatory muscles in patients with bruxism.

## Materials and methods

### Design

A randomized controlled study with three groups was conducted to examine the effect of deep-stripping massage on sleep quality, jaw mobility, or PPT of the masticatory muscles in patients with sleep bruxism.

### Participants

**Figure 1:**
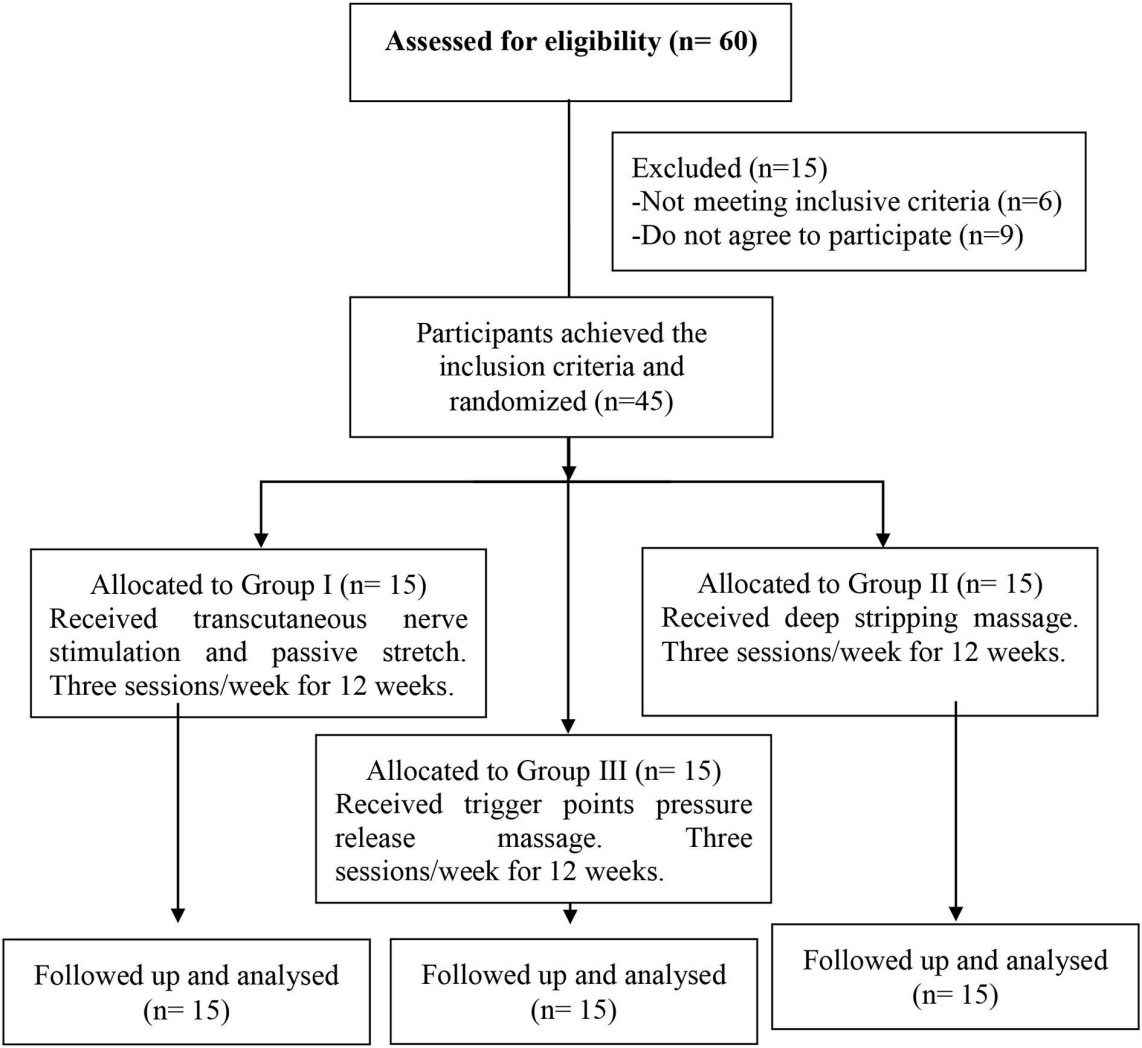
Flow of patients through the trial.

Forty-five patients diagnosed with sleep bruxism participated in the study. The patients had to meet the minimum diagnostic criteria for sleep bruxism, which involved complaining of tooth clenching or grinding during sleep, sounds accompanied by bruxism or discomfort in jaw muscles, and abnormal tooth wear (10). Patients were randomly assigned to three groups (Group I, Group II, and Group III). To ensure the random assignment of the patients to the tested groups, we used a computer-generated randomized table to implement randomization using the SPSS program (version 24 for Windows). Each participant had one identification number, which was used to assign participants to three groups equal in number (*n* = 15). Numbered index cards were secured in opaque envelopes, and a blinded researcher opened the sealed envelope and allocated the patients according to their groups. There were no dropouts among the patients throughout the study (Figure 1). The research protocol was approved by Cairo University's Supreme Council of Postgraduate Studies and Research and Human Research Ethics Committee (P.T.REC/012/001936). Written informed consent was obtained from all participants. This study conformed to all Consortium guidelines and is registered at clinicaltrials.gov (Identifier: NCT03753529).

### Eligibility criteria

A dentist referred patients to the outpatient clinic at the Faculty of Dentists, Cairo University. To be included in the study, patients had to be between 18 and 40 years old, had had chronic sleep bruxism for at least 3 months, and had to be diagnosed with myofascial temporomandibular disorders according to the Research Diagnostic Criteria for TMD: pain in the front of the ear, the temple, and the jaw improved with jaw movement, and pain and tenderness to palpation in the temporalis and masseter muscles (11). Additionally, patients had to have certain diagnoses of active myofascial trigger points in both the masseter and temporalis muscles, characterized by a palpable, hypersensitive taut band in the muscle, a local twitch response that increased with palpation of the tight band, and recurring pain from the myofascial trigger points. Moreover, the patients were to have a pain score of >30 on a 100 mm visual analog scale at the initial evaluation (9, 11, 12). Patients were excluded from the study if they had any past trauma or fracture of the mandible or TMJ, any neurological, systematic joint, or muscle disease, or previous physical therapy treatment for sleep bruxism or TMJ during the last month (12).

### Outcome measure

The measures below were collected by the same examiner before and after 6 weeks, during which each group received the determined intervention:

*Pittsburgh Quality Sleep Index (PQSI):* This index was used to assess the sleep quality in each group before and after 6 weeks. The PSQI consisted of 19 self-reported inquiries merged into seven subitems: subjective sleep quality, latency, duration, habitual efficiency, disturbances, usage of sleep drugs, and daytime dysfunction. Each item was rated from zero to three, with higher scores indicating poorer sleep quality. The seven items were then computed to give a single score ranging from zero to 21, with higher scoring indicating lower sleep quality (13). The PSQI was found to be valid and reliable in the evaluation of self-reported sleep problems (14).

*Jaw range of motion (ROM):* A vernier caliper was used to assess jaw opening, lateral movements, protrusion, and retraction ROM while sitting. The Vernier caliper is a reliable tool for evaluating jaw movements, with excellent intrasession and intersession reliability and at least moderately accurate intraclass correlation coefficients (15).

*Pressure pain threshold (PPT):* PPT was measured in the temporalis, masseter, lateral pterygoid, and digastric muscles using an Egyptian pressure algometer. The Egyptian algometer is a valid and reliable instrument for quantifying PPT in patients with bruxism with high intraclass correlation coefficients (16). First, the Egyptian algometer was started and calibrated to determine PPT. The rubber tip of the Egyptian algometer was then placed on the marked myofascial trigger point and was held vertically to the muscle belly. The examiner then increased the pressure on the selected point, and the patient was instructed to indicate when the feeling of pressure changed to pain or discomfort. As soon as the patients started to perceive pain, the pressure was stopped, and the reading was recorded (16).

### Procedures

Procedures were explained to the patients upon arrival, and informed consent was obtained. The informed consent was approved by Cairo University's Supreme Council of Postgraduate Studies and Research and Human Research Ethics Committee (P.T.REC/012/001936). Afterward, the PSQI was collected from each patient. TMJ range of motion (opening, protraction, retraction, and right and left lateral movements) and PPT were tested while sitting using a Vernier caliper and an Egyptian pressure algometer, respectively. Patients were tested two times, with 6 weeks in between. During this period, patients in Group I received transcutaneous nerve stimulation and passive stretching; those in Group II received deep-stripping massage, and those in Group III received trigger-point pressure release massage twice a week.

#### Group I intervention

Patients in Group I received transcutaneous electrical nerve stimulation while sitting with the upper limb supported, followed by a passive stretch of the masticatory muscles. After the overlying skin was cleaned with alcohol, electrodes were placed on the masseter and anterior temporalis muscles. TENS was applied for 20 min with a frequency of 150 Hz, a pulse width of 20 μs, and up to 50% modulation. Afterward, patients in Group I received passive stretching of the masticatory muscles. Each stretch was held for 20 s in a position of mild discomfort and repeated three times.

#### Group II intervention

Patients in Group II were administered deep-stripping massage to the target muscles while maintaining a relaxed position. The patient was positioned in a comfortable and completely lengthened position without pain or residual slack in the muscle. Both hands' thumbs or fingers were then placed in a way that trapped a taut band between them beyond the trigger point of the taut band. The pressure was then applied along the length of the taut band to elongate the sarcomeres of the shortened muscle. The deep-stripping massage should be applied along the length of the rest of the taut band beyond the trigger point to the attachment of the taut band (9). The next stripping massage should go in the other direction on the same taut band to release the shortened sarcomeres. The deep-stripping massage was repeated until the tenderness was reduced (9).

#### Group III intervention

Patients in Group III had received a pressure release massage on the active trigger points in the masseter and temporalis muscles. The treating therapist lengthened the muscle and then gradually increased pressure on the active trigger point until a definite increase in tissue resistance was felt. A patient may feel some discomfort or pain at that point. The applied pressure on the active trigger points was maintained until the treating therapist felt the relief of tissue tension under the finger or the patient demonstrated a considerable decrease in pain. This procedure was repeated several times until the applied pressure provoked no pain or little discomfort (17).

### Sample size estimation

The sample size was determined a priori using G^*^ power (version 3.1.9.2). The calculation was based on the F test, the type I error rate was set at 5% (alpha-level 0.05), and the effect size of 0.53 of the main outcome variable (PSQI) was obtained from a pilot study performed on five participants in each group. The type II error rate was set at 90% power.

### Statistical analysis

Statistical analysis was conducted using the Statistical Package for Social Science (SPSS) version 23 for Windows. Initially, data were screened for normality assumption using the Kolmogorov-Smirnov and Shapiro–Wilks normality tests with (*p*-value > 0.05). The data were also tested for extreme scores, significant skewness, and kurtosis. Moreover, data were screened for homogeneity of variance assumption within Levene's test (*p* > 0.05). Parametric data analysis was conducted as soon as the data were found not to violate the normality and homogeneity of variance assumptions. Accordingly, 3 × 2 mixed-design MANOVA was conducted to compare the tested variables among the three tested groups in each of the pre- and post-test conditions and to compare these conditions for the tested variables in each tested group. The initial alpha level was set at 0.05.

## Results

**Figure 2:**
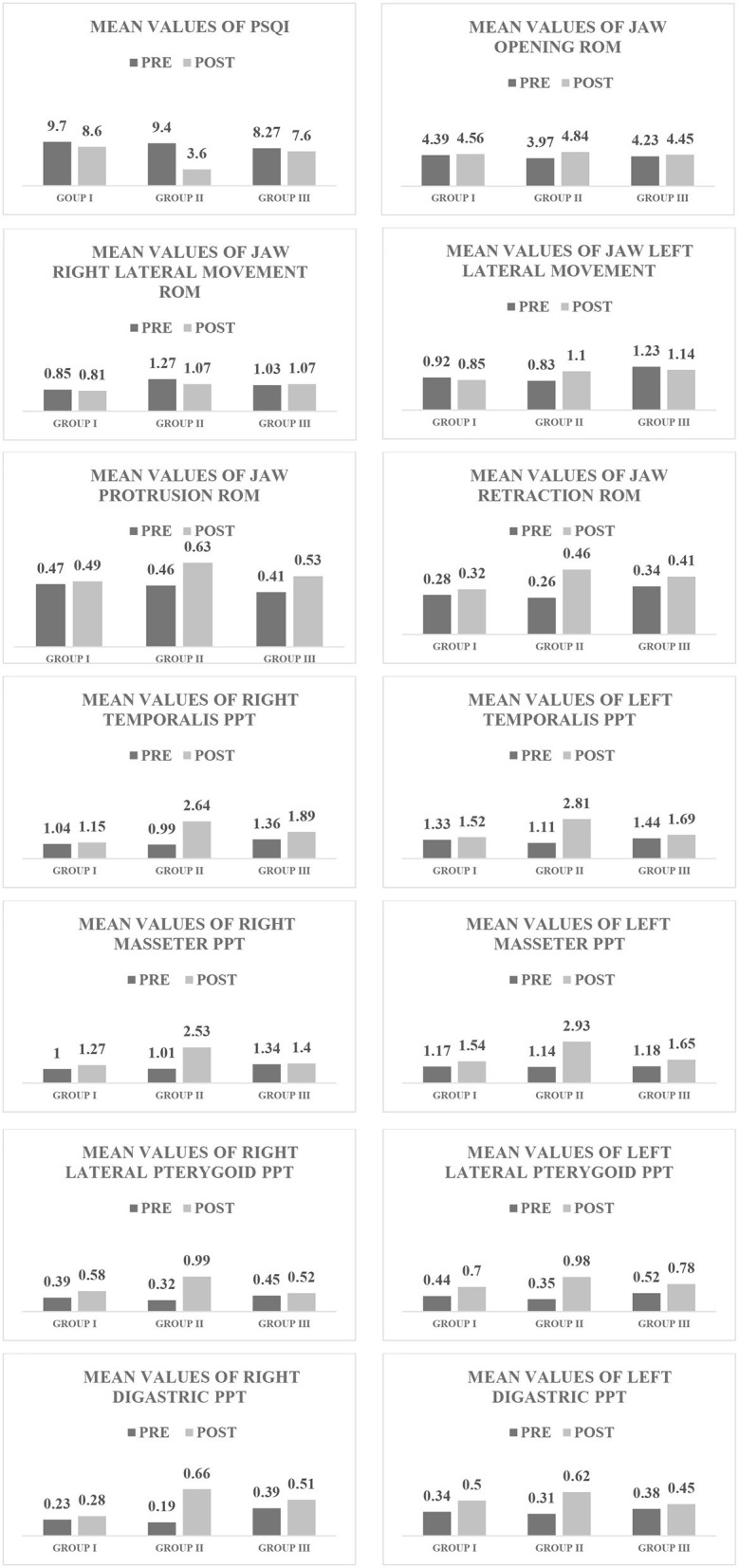
Mean PSQI, jaw ROM, and PPT values in each group in the “pre” and “post” test conditions.

As indicated by the one-way analysis of the variance, there were no significant differences (*p* > 0.05) in the mean values of weight, height, and body mass index among groups (Table 1). Additionally, there was no significant difference (*p* > 0.05) in sex distribution among groups, as indicated by the Chi-squared test (Table 1). The 3 × 2 mixed-design MANOVA with the subsequent multiple pairwise comparison tests revealed that there were significant improvements (*p* < 0.05 Tables 2, 3, Figure 2) in the mean values of the PSQI, ROM of jaw opening, jaw left lateral movement, jaw protrusion, and jaw retraction in the post-test condition in Group II compared with Group I and Group III. Moreover, there were significant improvements (*p* < 0.05 Tables 2, 3, Figure 2) in PPT of the right temporalis, left temporalis, right masseter, left masseter, right lateral pterygoid, left lateral pterygoid, right digastric, and left digastric muscles in the post-test condition in Group II compared with Group I and Group III.

**Table 1:** Mean (SD) and one-way ANOVA for the participants' demographic data.

	**Group I (*N* = 15)**	**Group II (*N* = 15)**	**Group III (*N* = 15)**	**F-value**	**p-value**
	**X ±SD**	**X ±SD**	**X ±SD**		
Age (years)	28.67 ± 7.01	23.8 ± 3.32	24.33 ± 4.94	3.787	0.052
Weight (kg)	75.9 ± 16.66	71 ± 14.43	65.33 ± 9.68	2.172	0.127
Height (cm)	164.93 ± 7.94	165.6 ± 9.73	166.06 ± 9.02	0.061	0.941
BMI(Kg/m^2^)	27.67 ± 4.55	25.75 ± 4.06	23.95 ± 4.54	2.689	0.080
**Sex distribution**					
	**Group I**	**Group II**	**Group III**	**X** ^2^	**P-value**
Male	10 (66.7%)	10 (66.7%)	8 (53.3%)	0.756	0.685
Female	5 (33.3 %)	5 (33.3 %)	7 (46.7%)		

X, Mean; SD, Standard deviation; ANOVA, One-way analysis of variance; p-value, Probability value, X^2^, chi-square value.

**Table 2:** The 3 × 2 mixed-design Multivariate Analysis of Variance (MANOVA) for the tested variables at the two measuring periods among the tested groups.

**Source of variation**	**F-value**	**P-value**	**Partial eta square**
Groups	2.93	0.0001*	0.586
Measuring periods	13.413	0.0001*	0.866
Interaction	5.124	0.0001*	0.712

^*^Significant at P < 0.05.

**Table 3:** Mean (SD) of groups and mean (SD) within groups for the tested variables in the “pre” and “post” test conditions.

**Outcome measures**		**Group I (*N* = 15)**	**Group II (*N* = 15)**	**Group III (*N* = 15)**	**Between group P-value**		
					**G I Vs. II**	**G I Vs. III**	**G II Vs. III**
PSQI	Pre	9.7 ± 4.1	9.40 ± 3	8.27 ± 3	0.999	0.766	0.999
	Post	8.6 ± 2.8	3.6 ± 2.2	7.6 ± 3.29	0.0001*	0.999	0.001*
Within group P-value	0.113	0.0001*	0.346			
Jaw opening ROM	Pre	4.39 ± 0.88	3.97 ± 1	4.23 ± 0.68	0.554	0.999	0.999
	Post	4.56 ± 0.73	4.84 ± 0.49	4.45 ± 0.41	0.511	0.999	0.175
Within group P-value	0.365	0.0001*	0.233			
Jaw right lateral movement ROM	Pre	0.85 ± 0.25	1.27 ± 2.17	1.03 ± 0.38	0.99	0.99	0.99
	Post	0.81 ± 0.27	1.07 ± 0.14	1.07 ± 0.33	0.03*	0.03*	0.99
Within group P-value	0.89	0.54	0.9			
Jaw left lateral movement ROM	Pre	0.92 ± 0.4	0.83 ± 0.26	1.23 ± 0.47	0.99	0.098	0.052
	Post	0.85 ± 0.33	1.1 ± 0.29	1.14 ± 0.37	0.146	0.069	0.99
Within group P-value	0.045*	0.004*	0.29			
Jaw protrusion ROM	Pre	0.47 ± 0.22	0.46 ± 0.19	0.41 ± 0.1	0.99	0.93	0.99
	Post	0.49 ± 0.23	0.63 ± 0.27	0.53 ± 0.13	0.297	0.99	0.638
Within group P-value	0.64	0.0001*	0.007*			
Jaw retraction ROM	Pre	0.28 ± 0.19	0.26 ± 0.18	0.34 ± 0.18	0.99	0.99	0.699
	Post	0.32 ± 0.17	0.46 ± 0.21	0.41 ± 0.13	0.091	0.518	0.99
Within group P-value	0.396	0.0001*	0.160			
Right temporalis PPT	Pre	1.04 ± 0.68	0.99 ± 0.48	1.36 ± 0.61	0.056	0.459	0.137
	Post	1.15 ± 0.75	2.64 ± 0.87	1.89 ± 0.69	0.001*	0.036*	0.032*
Within group P-value	0.58	0.0001*	0.009*			
Left temporalis PPT	Pre	1.33 ± 0.96	1.11 ± 0.79	1.44 ± 0.73	0.99	0.99	0.83
	Post	1.52 ± 0.94	2.81 ± 0.95	1.69 ± 0.85	0.001*	0.99	0.005*
Within group P-value	0.42	0.0001*	0.29			
Right masseter PPT	Pre	1.00 ± 0.45	1.01 ± 0.61	1.34 ± 0.46	0.99	0.25	0.27
	Post	1.27 ± 0.51	2.53 ± 0.96	1.40 ± 0.37	0.001*	0.99	0.001*
Within group P-value	0.19	0.001*	0.76			
Left masseter PPT	Pre	1.17 ± 0.44	1.14 ± 0.83	1.18 ± 0.46	0.99	0.99	0.99
	Post	1.54 ± 0.61	2.93 ± 1.35	1.65 ± 0.46	0.001*	0.99	0.001*
Within group P-value	0.08	0.001*	0.03*			
Right lateral pterygoid PPT	Pre	0.39 ± 0.17	0.32 ± 0.24	0.45 ± 0.19	0.99	0.99	0.28
	Post	0.58 ± 0.28	0.99 ± 0.44	0.52 ± 0.23	0.004*	0.99	0.001*
Within group P-value	0.05*	0.001*	0.47			
Left lateral pterygoid PPT	Pre	0.44 ± 0.19	0.35 ± 0.19	0.52 ± 0.26	0.74	0.99	0.13
	Post	0.7 ± 0.21	0.98 ± 0.39	0.78 ± 0.55	0.19	0.99	0.52
Within group P-value	0.02*	0.001*	0.01*			
Right digastric PPT	Pre	0.23 ± 0.25	0.19 ± 0.29	0.39 ± 0.51	0.99	0.75	0.46
	Post	0.28 ± 0.30	0.66 ± 0.51	0.51 ± 0.56	0.11	0.60	0.99
Within group P-value	0.56	0.001*	0.18			
Left digastric PPT	Pre	0.34 ± 0.31	0.31 ± 0.38	0.38 ± 0.25	0.99	0.99	0.99
	Post	0.5 ± 0.39	0.62 ± 0.32	0.45 ± 0.29	0.94	0.99	0.54
Within group P-value	0.06	0.001*	0.34			

^*^Significant at P < 0.05; PSQI, Pittsburgh Sleep Quality Index; ROM, Range of motion; PPT, Pressure pain threshold; Between-group, Comparison among groups for PSQI, jaw ROM and PPT in each of the pre-test and post-test conditions; Within group, Comparison between the pre-test and post-test conditions for the tested variables in each of the tested groups.

## Discussion

To the best of the authors' knowledge, this is the first study that examined the effect of deep-stripping and trigger-point pressure release massage on the quality of sleep, jaw mobility, or PPT of the masticatory muscles in patients with chronic sleep bruxism. The findings of this study indicated that those treated with deep-stripping massage over 6 weeks had improved sleep quality and jaw mobility, or PPT, of the masticatory muscles compared with the trigger-point pressure release massage group (Group III). Additionally, these improvements were not observed in Group I, which received transcutaneous electric nerve stimulation and passive stretching. The improvement in sleep quality following treatment using deep-stripping massage was evident in a decrease in the PSQI score. At the initial testing, Group II and Group III reported similar PSQI scores (9.4 and 8.2). After 6 weeks, the PSQI score decreased by six points in Group II and one point in Group III.

Improvements in TMJ mobility following treatment with deep-stripping massage were evident by an increase in the degree of TMJ opening, left lateral movement, protrusion, and retraction. On the other hand, Group III, who received trigger-point pressure release massage for the same period, had improved jaw protrusion ROM only. The increase in jaw mobility in the deep-stripping massage group was observed, along with a significant increase in PPT of the masticatory muscles. The deep-stripping massage group showed much more improvement in PPT in the post-test condition (8 muscles from 8) compared with the trigger-point pressure release massage group (3 muscles from 8) and Group I (2 muscles from 8). This significant improvement in PPT after applying deep-stripping massage may be attributed to mechanical pressure. The deep-stripping massage exerts forces on mechanoreceptors and proprioceptors (18). More specifically, deep-stripping massage can provide analgesic effects through the ascending pain inhibitory system. It can alter the transmission of ascending nociceptive information and modulate pain perception (19). Moreover, deep-stripping massage may prevent the unnecessary firing of muscle spindles, decrease trigger point-induced muscle spasms, decrease pain, and improve joint mobility (19).

These findings are consistent with those reported by Blasco-Bonora and Martín-Pintado-Zugasti (12). They examined the effects of deep dry needling of myofascial trigger points of the masseter and temporalis muscles on pain, PPT, jaw opening, and TMD-related disability in patients with sleep bruxism and myofascial TMD. The masseter and temporalis muscles were repeatedly perforated with a solid stainless-steel filament needle until no more local twitch responses were elicited. PPT, jaw opening, and TMD-related disability were assessed before, immediately after, and 1 week after treatment. They concluded that the treatment had adequately improved pain, PPT, jaw opening, and TMD-related disability. It should be noted that they used dry needling only once on the tested muscles. However, in our study, each program was performed three times a week for 6 weeks.

Similar findings were obtained using occlusal splints for TMD and bruxism (20). Although it is not well-known how occlusal splints treat bruxism and TMD, occlusal splints possibly decrease myofascial pain and improve jaw mobility, improving the quality of life in patients with bruxism (21–23). Moreover, this study's findings are consistent with those of a previous study (24) assessing the effects of two massage techniques on the upper trapezius myofascial trigger point. In a sample of 30 patients treated using ischemic compression and muscle energy technique over a period of 4 weeks, improvement in pain and cervical side flexion range of motion was noted in both groups. However, the muscle energy technique was more effective in reducing pain and improving cervical side flexion range of motion. In the same context, the significant improvement in PPT is in accordance with that reported by Albertin et al. (25). They found that manual therapy with transversal and circular movements significantly improved pain in the masseter muscle without any significant change in the electrical activity of the muscle.

Using surface electromyography of the masseter and temporalis muscles, Nascimento et al. (26) approved the long-term effects of using an occlusal splint in patients with sleep bruxism and signs and symptoms of TMD. They declared that the electrical activity of the masseter and temporalis muscles did not change after 60 days of occlusal splint use. However, there was a significant decrease in TMD signs and symptoms using occlusal splints. Similar results were obtained by Harada et al. (27). They investigated the effects of stabilization splints and platelet splints on the electrical activity of the right masseter muscle in 16 patients with sleep bruxism. They declared that both splints decreased the masseter muscle's electromyographic activity. However, this improvement was transient. Similarly, electromyographic activity did not significantly improve by using occlusal splints over a period of 12 weeks in patients with chronic myofascial temporomandibular pain (28).

The authors of this study thought that deep-stripping massage of the masticatory muscles would improve the sleep quality, jaw mobility, and pressure pain threshold of the masticatory muscles in patients with sleep bruxism. The use of deep-stripping massage on the muscles of mastication would increase the functionality of these muscles, improve sleep quality and quality of life, and reduce the emotional stress accompanied by sleep bruxism. Only patients with strict inclusion criteria indicating chronic sleep bruxism were included in the study. It is yet to be determined if similar results would apply to patients who present with different types of bruxism. Additionally, clinical follow-up of patients after treatment was not performed, which limited our ability to prove the long-term effects of such treatments. Therefore, future studies should include a long-term follow-up to investigate the long-term effect of deep-stripping massage on bruxism.

## Conclusion

PSQI, jaw mobility, and PPT of the masticatory muscles were tested before and after each intervention. The results of the current study revealed that deep-stripping massage was more effective for improving PSQI, jaw mobility, or PPT of the masticatory muscles in patients with chronic sleep bruxism than trigger-point pressure release massage and traditional treatment techniques.

## Data availability statement

The raw data supporting the conclusions of this article will be made available by the authors, without undue reservation.

## Ethics statement

The studies involving human participants were reviewed and approved by the Cairo University's Supreme Council of Postgraduate Studies and Research and Human Research Ethics Committee (P.T.REC/012/001936). Written informed consent was obtained from all participants. The patients/participants provided their written informed consent to participate in this study.

## Author contributions

ME-G, MI, and EH: conception and design. ME-G, MI, EH, NN, BA, GK, and HH: acquisition, analysis, and interpretation of the data, responsible for all parts of the work, and ensuring that any concerns about its accuracy or integrity are properly examined and addressed. MI, NN, BA, GK, and HH: drafting and revising the paper critically for important intellectual content. All authors contributed to the article and approved the submitted version.
